# A Meta-analysis of the Effectiveness of Albendazole Compared with Metronidazole as Treatments for Infections with *Giardia duodenalis*

**DOI:** 10.1371/journal.pntd.0000682

**Published:** 2010-05-11

**Authors:** Shahram Solaymani-Mohammadi, Jeanine M. Genkinger, Christopher A. Loffredo, Steven M. Singer

**Affiliations:** 1 Department of Biology, Georgetown University, Washington, D. C., United States of America; 2 Department of Oncology, Lombardi Cancer Center, Georgetown University Medical Center, Washington, D. C., United States of America; Swiss Tropical Institute, Switzerland

## Abstract

**Background:**

Metronidazole is the most commonly used drug for the treatment of giardiasis in humans. In spite of its therapeutic efficacy for giardiasis, low patient compliance, especially in children, side effects, and the emergence of metronidazole-resistant strains may restrict its use. Albendazole has been used to treat *Giardia duodenalis* infections in recent years. However, efficacy studies *in vivo* and *in vitro* have produced diverse results as to its effectiveness. A moderately benign side effect profile, combined with established efficacy against many helminths, renders it promising for treatment of giardiasis in humans.

**Methodology and Principal Findings:**

We performed a search in the PubMed, Scopus, EMBASE, the ISI Web of Science, LILIACS, and Cochrane Controlled Trials Register for trials published before February 2010 as well as in references of relevant research and review articles. Eight randomized clinical trials (including 900 patients) comparing the effectiveness of albendazole with that of metronidazole were included in meta-analysis. After extracting and validating the data, the pooled risk ratio (RR) was calculated using an inverse-variance random-effects model. Albendazole was found to be equally as effective as metronidazole in the treatment of giardiasis in humans (RR 0.97; 95% CI, 0.93, 1.01). In addition, safety analysis suggested that patients treated with albendazole had a lower risk of adverse effects compared with those who received metronidazole (RR 0.36; 95% CI, 0.10, 1.34), but limitations of the sample size precluded a definite conclusion.

**Conclusions/Significance:**

The effectiveness of albendazole, when given as a single dose of 400 mg/day for 5 days, was comparable to that of metronidazole. Patients treated with albendazole tended to have fewer side effects compared with those who took metronidazole. Given the safety, effectiveness, and low costs of albendazole, this drug could be potentially used as an alternative and/or a replacement for the existing metronidazole therapy protocols in the treatment of giardiasis in humans.

## Author Summary

Giardiasis is one of the most common intestinal protozoal infections worldwide. Although metronidazole is the most common drug used to treat giardiasis in humans, its use is associated with a variety of side effects. Poor compliance and the emergence of metronidazole-resistant strains may restrict use of the drug. Albendazole is an orally administered broad-spectrum anthelmintic agent. The use of albendazole has fewer side effects than metronidazole. The anthelmintic has been used against *Giardia duodenalis* both *in vivo* and *in vitro* with different results. However, the current meta-analysis assessed the effectiveness and safety of albendazole compared with metronidazole for the treatment of giardiasis in humans. After searching different databases, eight comparative randomized clinical trials, including 900 patients, met our criteria and were selected for the current meta-analysis. Results showed that albendazole was as effective as metronidazole for the treatment of giardiasis in humans and people receiving the drug tended to have fewer side effects compared with those who received metronidazole. Given the safety, effectiveness, and low costs of albendazole, this drug may be considered a potential alternative and/or a replacement for the existing widely used metronidazole in the treatment of giardiasis in humans.

## Introduction

Giardiasis in humans, caused by the protozoan parasite *Giardia duodenalis* (syn. *G. lamblia*, *G. intestinalis*), is a common parasitic disease [1]. The prevalence of infection is commonly between 2–5% in the developed world and 20–30% in the developing and underdeveloped countries [2]. Infection is initiated by ingestion of cysts in contaminated drinking water and/or contaminated food [1]. Ingested cysts release trophozoites which colonize and replicate in the small intestine of the new host. *G. duodenalis* does not invade the epithelial or deeper layers of the mucosa and propagation takes place on the epithelial surface [3]. The outcomes of *Giardia* infections vary significantly and the majority of infections are self-limiting. Clinical manifestations range from a relatively asymptomatic phase marked by mild nutrient malabsorption, to an ephemeral or persistent acute stage, with steatorrhea, intermittent diarrhea, vomiting, malabsorption syndrome and weight loss, or to a subacute chronic phase that can mimic gallbladder or peptic ulcer disease [4], [5]. Infections in immunocompetent individuals are generally self-limited, suggesting the existence of effective host defense mechanisms against the parasite [6]. Different diagnostic methods are employed for the diagnosis of human giardiasis of which the most insensitive method, direct stool microscopy is used routinely in developing countries where the disease is endemic [5], [7].

Existing chemotherapy protocols recommend that patients should be treated if the parasite is found, irrespective of the presence or absence of acute symptoms [8]. However, some investigators question the usefulness of chemotherapy in infected people in endemic areas due to the extremely high rate of reinfection, as high as 90% in some studies [9], [10]. Treatment preferences vary among clinicians and in different locations. Several synthetic compounds (including metronidazole and other nitroimidazole derivates such as albendazole, mebendazole, furazolidone, tinidazole, ornidazole) are used in the treatment of giardiasis in humans. A single dose of tinidazole (2.0 g) has been shown to have a clinical efficacy of 80–100% in different clinical trials [11], [12] while the compliance is improved compared with other giardiasis treatments. However, the high cost of tinidazole may restrict its use in mass chemotherapy campaigns [13] in developing and underdeveloped countries (e.g. $18 to $32 for a single-dose of 2 g for the treatment of trichomoniasis). The most widely used treatment protocols employ metronidazole given 3 times per day for 3–5 days [8], [14], [15], [16]. Metronidazole is typically administered in doses of 250 mg 3 times a day for 5–7 days for adults and 15 mg/kg 3 times a day for 5–7 days in children. However, albendazole is typically given as a single dose of 400 mg/day for 3–5 days. In recent years, therapeutic failure of metronidazole, the first-line drug of choice in giardiasis in humans, has increasingly been reported from all around the world [17]. Metronidazole is prescribed widely for a wide range of non-parasitic infectious diseases; overusing metronidazole as a treatment option for parasitic infections may increase the chances of the development of clinically drug-resistant strains of *Helicobacter pylori*, an important cause of gastric cancer in humans [18]. Low compliance of patients with the current metronidazole therapy protocols, the emergence of the metronidazole-resistant strains of the parasite and other pathogens, and rapid reinfection of treated patients in the endemic areas are additional reasons for considering alternative therapies [19].

Treatment compliance is a key factor affecting the outcome of giardiasis. However, compliance has been neglected in the literature [20], and is therefore not part of the current analysis. In one report on metronidazole use in patients with giardiasis, treatment compliance was extremely poor because of missed doses, spillage, inaccurate measuring implements, and poor adherence to the prescribed frequency and duration of medication [21]. Common adverse reactions frequently reported with metronidazole include metallic taste, nausea, vomiting, diarrhea, and epigastric discomfort [20]. Moreover, its activity against the host's normal intestinal microflora; its contraindication for children, pregnant and breastfeeding women; and its carcinogenic and tumorigenic properties in animal models make it less than optimal for widespread use [8]. Finding safer drugs with less toxicity and more effective therapeutic properties and developing novel protocols (e.g fewer doses and shortened duration) to maximize the effects of existing drugs are, therefore, crucial for the field.

Albendazole has been used extensively for the treatment of a wide range of helminth parasites including hookworms, *Ascaris lumbricoides*, *Trichuris trichiura*, *Echinococcus sp.* [22] and *Taenia sp.* [23] with few side effects (reviewed by Keiser and Utzinger [24]). The mechanism of action of albendazole differs from that of metronidazole. While metronidazole affects electron transport of the parasite [25], it is believed that albendazole exerts its anti-giardial effects by interaction with tubulin of the *Giardia* cytoskeleton [26]. Albendazole also has overt giardiacidal activity *in vitro* [27], as well as being able to resolve infections in a mouse model of *G. duodenalis* infection [19], [26]. Using albendazole against giardiasis in humans could potentially augment mass treatment programs, which are part of helminth control campaigns, since most patients with *Giardia* are probably co-infected with other parasitic agents. Altogether, the evidence suggests that albendazole could be considered as a potential anti-giardial agent. Its lower toxicity, its relative insolubility and poor absorption from the gut, and its lack of significant effects on the intestinal microflora could make albendazole an ideal substitute for metronidazole. The aims of the current meta-analysis, therefore, were first to address the effectiveness and second to assess the safety of albendazole compared with metronidazole for the treatment of giardiasis in humans.

## Methods

### Data source and study selection

A literature search of the PubMed database (1966–February 2010), Scopus, EMBASE, the Cochrane Controlled Trials Register (issue 4, 2009), LILIACS and the ISI Web of Science for trials published before February 2010 was performed. The literature search used the following terms: “giardiasis”, “metronidazole”, and “albendazole.” The abstracts of all selected articles were read to identify the potentially eligible articles. A manual search was performed systematically using the authors' reference files and reference lists from original communications, selected books and review articles [8], [28]. Language restriction was not applied. The contents of abstracts or full-text manuscripts identified during our literature search were reviewed to determine whether they met the criteria for inclusion. For inclusion, a study had to allocate the study participants randomly to study groups (a prospective randomized clinical trial). Included studies had to compare the effectiveness of albendazole with that of metronidazole in the treatment of giardiasis.

**Figure 1:**
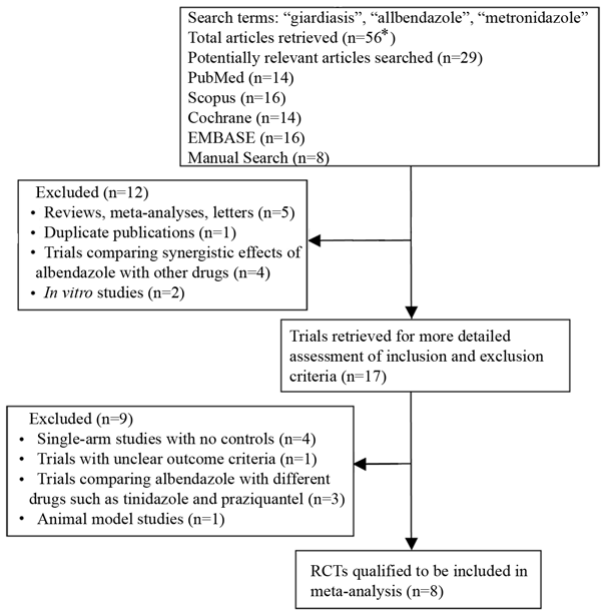
Flow diagram deciphering the article selection process for this meta-analysis study. Individual searches do not add up to 56 as some of the same articles were retrieved by multiple search engines.

Figure 1 summarizes the trial selection process. Our search identified twenty-nine articles for further consideration, of which only eight articles met the inclusion criteria. Major reasons for exclusion of studies were duplicate publications from which only one article was selected [29], [30], animal models of infections [19], [26], studies of veterinary importance [31], studies *in vitro* [32], single-arm studies with no randomized control groups [33], studies lacking a comparison between the effectiveness of albendazole with metronidazole [34], review articles [8], [28], studies with no clear randomization allocation procedure [35], studies using albendazole and metronidazole analogues [36] as well as the studies showing the synergistic effects between albendazole and/or metronidazole with other drugs [37], [38]. Conference proceedings and unpublished data were also not included. Included articles compared the effectiveness of albendazole with that of metronidazole in the treatment of giardiasis [29], [39]–[45]. Together these articles followed 900 patients presenting with symptomatic and/or asymptomatic *G. duodenalis* infections. Among these 900 treated patients, 452 (50.2%) individuals were treated with albendazole whereas 448 (49.8%) received metronidazole.

### Data extraction and analysis

Data were extracted independently by two reviewers (SSM and SMS) from the eight randomized controlled trials [Table 1 and Table 2]. Discrepancies were resolved by discussion. Study characteristics recorded were as follows: 1) first author's name, year of publication and country of origin; 2) description of the population; 3) number of participants; 4) age and sex distribution of the participants; 5) number of participants in each arm; 6) clinical profile (symptomatic, asymptomatic infections); 7) the follow-up period; 8) the outcome measure; 9) study design; 10) type and dosage of the drugs; and 11) effectiveness range.

**Table 1:** Characteristics of the randomized controlled trials included in the meta-analysis.

Author, Year (Country)	Study Design	No. of Randomized Participants	Age (yr)	Disease Characteristics	Anti-giardial Drug Regimens (No. of Participants)	Efficacy
Alizadeh, 2006 (Iran) [39]	Open-label*, RCT	120	2–53	Symptomatic	Albendazole, 400 mg/d for 5d (60)	Albendazole (90%)
	Two parallel arms				Metronidazole, 250 mg tid for 5d (60)	Metronidazole (76.7%)
Yereli, 2004 (Turkey) [41]	Open-label, RCT	107	3–15	Symptomatic	Albendazole, 10 mg/kg sid for 5d (52)	Albendazole (90.4%)
	Two parallel arms			Asymptomatic	Metronidazole, 20 mg/kg tid for 7d (57)	Metronidazole (89.1%)
Karabay, 2004 (Turkey) [40]	Open-label, RCT	57	41±12¥	Symptomatic	Albendazole, 400 mg/d for 5d (28)	Albendazole (96.4%)
	Two parallel arms		38±14‡		Metronidazole, 500 mg tid for 5d (29)	Metronidazole (100%)
Rodríguez-García, 1996 [44]	Open-label, RCT	49	3–12	Symptomatic	Albendazole, 200 mg tid for 5 d (27)	Albendazole (77%)
	Two parallel arms			Asymptomatic	Metronidazole, 30 mg/kg tid for 5 d (22)	Metronidazole (72.7%)
Misra, 1995 (India) [29]	Open-label, RCT	34	2–12	Symptomatic	Albendazole, 400 mg/d for 5d (18)	Albendazole (100%)
	Two parallel arms				Metronidazole, 7.5 mg/kg tid for 5d (16)	Metronidazole (100%)
Romero-Cabello, 1995 (Mexico) [42]	Open-label, RCT	100	4–11	Symptomatic	Albendazole, 400 mg/d for 5d (50)	Albendazole (94%)
	Two parallel arms			Asymptomatic	Metronidazole, 7.5 mg/kg tid for 5d (50)	Metronidazole (98%)
Dutta, 1994 (India) [45]	Open-label&, RCT	150	2–10	N.S.#	Albendazole, 400 mg as a single dose (75)	Albendazole (97%)
	Multicenter, Two parallel arms				Metronidazole, 22.5 mg/kg tid for 5d (75)	Metronidazole (97%)
Hall, 1993a (Bangladesh) [43]	Open-label§, RCT	283	5–10	N.S.€	Albendazole, 400 mg sid for 3d (116)	Albendazole (87.8%)
	Three parallel arms in each trial				Metronidazole, 125 mg tid for 5d (115)	Metronidazole (98.7%)
Hall, 1993b (Bangladesh) [43]	Open-label§, RCT	283	5–10	N.S.€	Albendazole, 400 mg sid for 5d (115)	Albendazole (94.1%)
	Three parallel arms in each trial				Metronidazole, 125 mg tid for 5d (115)	Metronidazole (100%)

Abbreviations: N.S., Not Stated; s.i.d., once a day; t.i.d., three times a day; RCT, randomized clinical trial.

¥Albendazole group.

‡Metronidazole group.

#The included patients were probably symptomatic individuals referred to three hospitals in India.

€Initially 768 children were screened in an urban slum in Dhaka from which 678 children were found to be infected with *Giradia*. The infected children were probably asymptomatic cyst-passers.

*The person who performed the stool microscopy was blinded to the treatments regimens.

&The stool sample examiner was blinded to the treatment regimens.

§Stool examination was done blinded to the treatment status of the patient.

**Table 2:** Follow-up, outcomes assessment and relative risk in the trials included in the meta-analysis.

Author, Year (Country)	Follow-up Duration (days)	Outcome Measure	Parasitological Methods Used for Assessing The Outcomes	Relative Risk® (95% Confidence Interval)	Comments
Alizadeh, 2006 (Iran) [39]	10	P	Direct iodine-stained wet preparations	1.17 (1.00, 1.38)	15 patients from the albendazole group and 9 patients from the metronidazole group failed to complete the course of medication
Yereli, 2004 (Turkey) [41]	14	P	Direct saline-lugol wet preparationsFormalin-acetate concentration methodTrichrome staining methods	1.01 (0.89, 1.15)	No side effects were reported for patients treated with either albendazole or metronidazole during therapy
Karabay, 2004 (Turkey) [40]	15	P	Direct iodine-stained wet preparations	0.96 (0.88, 1.06)	Individuals with pre-existing conditions such as pregnant women were excluded from the study
Rodríguez-García, 1996 [44]	14	P	Faust's concentration method	1.07 (0.77, 1.48)	-
Misra, 1995 (India) [29]	21	P, C	Direct saline wet preparationsFormalin-ether concentration method	0.95 (0.81, 1.11)	Only 18/32 and 16/32 children in the albendazole and metronidazole groups, respectively finished the study. At the end of follow-up period, the *Giardia* cysts were found in the stool of a child in the albendazole group
Romero-Cabello, 1995 (Mexico) [42]	21	P, C	Direct saline wet preparationsFlotation methods	0.96 (0.89, 1.04)	-
Dutta, 1994 (India) [45]	21	P, C	Not clearly stated	1.00 (0.95, 1.05)	Children having grade I and II malnutrition, acute febrile disease and those who had received medication for giardiasis were excluded from the study
Hall, 1993a (Bangladesh) [43]	10	P	Direct saline wet preparationFormalin-ether concentration method	0.89 (0.81, 0.97)	In Hall, 1993 a, b, the authors calculated the treatment efficacy rates in patients with first infections vs. reinfection separately. To make the results comparable to what was done previously [Zaat et.al, 1997], we included the first-infection cases, excluding the single-dose regimens and reinfection cases
Hall, 1993b (Bangladesh) [43]	10	P	Direct saline wet preparationFormalin-ether concentration method	0.94 (0.88, 1.01)	See above.

Abbreviations: P, parasitological; C, clinical cure.

®Relative risks were calculated separately for each study outcome using the software Rev Man5.

The primary outcome measure was parasitological cure defined as the absence of parasites (trophozoites and/or cysts) in feces at the end of the treatment in at least two consecutive stool microscopy examinations. Parasitological cure was considered necessary in order to evaluate the effectiveness of the treatment. The secondary outcome measure, clinical cure, was defined as the global improvement of clinical symptoms, such as diarrhea, nausea/vomiting, transient abdominal pain and loss of appetite, at the end of the follow-up period.

### Assessment of study quality

The quality of included reports was compared using the Jadad score which examines whether there is randomization, blinding, and information on dropouts/withdrawals from the study [46]. It also evaluates the appropriateness of randomization and blinding, if present. The quality scale ranges from 0 to 5 points with a low-quality report earning score of 2 or less. A study with a Jadad score ≥3 is considered to be of ample quality. The quality of parasitological diagnostic methods was assessed by the scoring system utilized by Zaat et al. [47]. This method evaluates whether techniques are sufficiently described and are adequate. Moreover, this method evaluates the reproducibility of the parasitological examinations and the level of inter-observer variation among methods [Table 3].

**Table 3:** Internal validity (methodological and parasitological) of included trials.

Trial	Methodological Assessment (Jadad Score)§				Parasitological Assessment¥				
	Randomized€?	Double-Blinded?®	A Description of Withdrawals or Dropouts?	Total Jadad Score	Description	Adequate	Repeated	Interobserver	Total
Alizadeh, 2006 [39]	1	0	1	2	2	0	3	0	5
Yereli, 2004 [41]	1	0	1	2	2	8	3	0	13
Karabay, 2004 [40]	1	0	1	2	2	0	3	0	5
Rodríguez-García, 1996 [44]	1	0	0	1	2	3	3	0	8
Misra, 1995 [29]	1	0	1	2	2	3	3	0	8
Romero-Cabello, 1995 [42]	1	0	1	2	2	3	3	0	8
Dutta, 1994 [45]	1	0	1	2	2	0	3	0	5
Hall, 1993a,b [43]	2	0	1	3	2	4	3	0	9

§Range 0–5 (5 exemplifies articles with the highest quality).

¥Range 0–15, (15 indicates most optimal diagnostic procedure employed).

€Represents generation of allocation sequence.

®Represents allocation concealment.

### Sensitivity analysis

Three different methods were employed to perform sensitivity analysis of these trials. We first excluded the trial in which the parasitological method employed was not clearly described [45]. Second, the trials that utilized the most insensitive diagnostic methods, i.e. direct stool microscopy, alone were excluded [39], [40]. Finally, we excluded a trial that used the most sensitive parasitological methods (three methods at the same time) [41], and compared the results with the remaining trials which used two parasitological methods.

### Data synthesis, statistical analysis

We identified eight randomized, controlled trials that reported data on the comparison of the effectiveness of albendazole with metronidazole in the treatment of giardiasis in humans. The inconsistency across trials was calculated using the I^2^ statistic; results range between 0% (i.e., no observed heterogeneity) and 100% [48]. High values reflect increasing heterogeneity. Publication bias was assessed by means of funnel plots [49]. Relative risks (RRs) were calculated for each study outcome separately based on information presented in articles (i.e. the percentage of people exhibiting parasitological cure in both groups relative to the percentage of people continuing to shed cysts during the follow up period); the pooled RRs and 95% confidence intervals (CIs) were estimated by using the inverse-variance random-effects method [50]. Although there is no standard description, an I^2^ statistic greater than 20% suggests heterogeneity while an I^2^ statistic greater than 50% usually is considered to represent significant heterogeneity [48]. The statistical package Review Manager Software 5 (Cochrane Collaboration, Oxford, UK) was used for analyzing the data.

## Results

Table 1 and Table 2 summarize the characteristics of the randomized clinical trails (RCTs) included in the meta-analysis. Studies were conducted in areas that are endemic for giardiasis in humans, including Iran [39], Turkey [40], [41], Mexico [42], [44], India [29], [45] and Bangladesh [43]. Only one study [43] was rated as having good methodological quality based on a Jadad score of 3 (see Table 3). However, because of the difficulty of comparing different treatment protocols, one would rarely expect to achieve a high Jadad score of 3 or greater. Only two studies [39], [43] had at least one blinded outcome measurement (parasitological cure); whereas the other 6 trials were open label randomized clinical trials (RCTs), allocating patients to albendazole and metronidazole groups randomly. Patients included in one of the groups in all trials were given albendazole. The dosages of albendazole ranged from 10 mg/kg sid for 5 days [41] to 400 mg/d for 5 days in most trials; lengths of therapy ranged from a single dose for 1 day to 5 days. Metronidazole dosage ranged from 22.5 mg/day [29] to 1500 mg/day [40], and the treatment course varied from 5 to 7 days. In six studies, subjects had symptomatic and/or asymptomatic giardiasis while the clinical status of patients in one study [45] was unclear. It is likely that the overwhelming majority, if not all, of the cases included in the study of Hall and Nahar [43] were asymptomatic cyst-passers, since the general population in an urban slum in Dhaka, Bangladesh was screened. The post-treatment follow-up differed across the studies from 10 days [39], [43] to 21 days [29], [42], [45]. Loss of follow-up did not occur in six studies whereas two articles [29], [39] reported withdrawals and/or dropouts. In one study [29], only 18/32 children in the albendazole group and 16/32 children in the metronidazole group finished the study, while in the other study, of 60 patients in each arm 15 from the albendazole group and 9 from the metronidazole group failed to complete the course of medication [39]. Side effects from metronidazole therapy did not appear to influence the treatment outcome, since low compliance in the latter study was reported to be due to difficulties in returning to the study clinic rather than to side effects of the treatments.

The included studies implemented different diagnostic procedures alone or in combination with other parasitological methods. As seen in Table 2, Yereli et al. [41] applied three different parasitological methods at the same time (a parasitological assessment score of 13 out of 15). Misra et al. [29], Romero-Cabello et al. [42], and Nahar and Hall [43] employed two different parasitological methods at the same time, Rodríguez-García et al. [44] used the Faust's concentration method whereas Alizadeh et al. [39] and Karabay et al. [40] utilized the least sensitive parasitological method, conventional direct stool microscopy, for measuring the outcomes. In all studies, the absence of detectable *G. duodenalis* trophozoites and/or cysts in the stool microscopy during the follow-up period was required to declare the patients cured; five studies measured the outcomes solely based on parasitological parameters [39], [40], [41], [43], [44], while three studies applied both parasitological and clinical parameters for measuring the outcomes [29], [42], [45].

**Figure 2:**
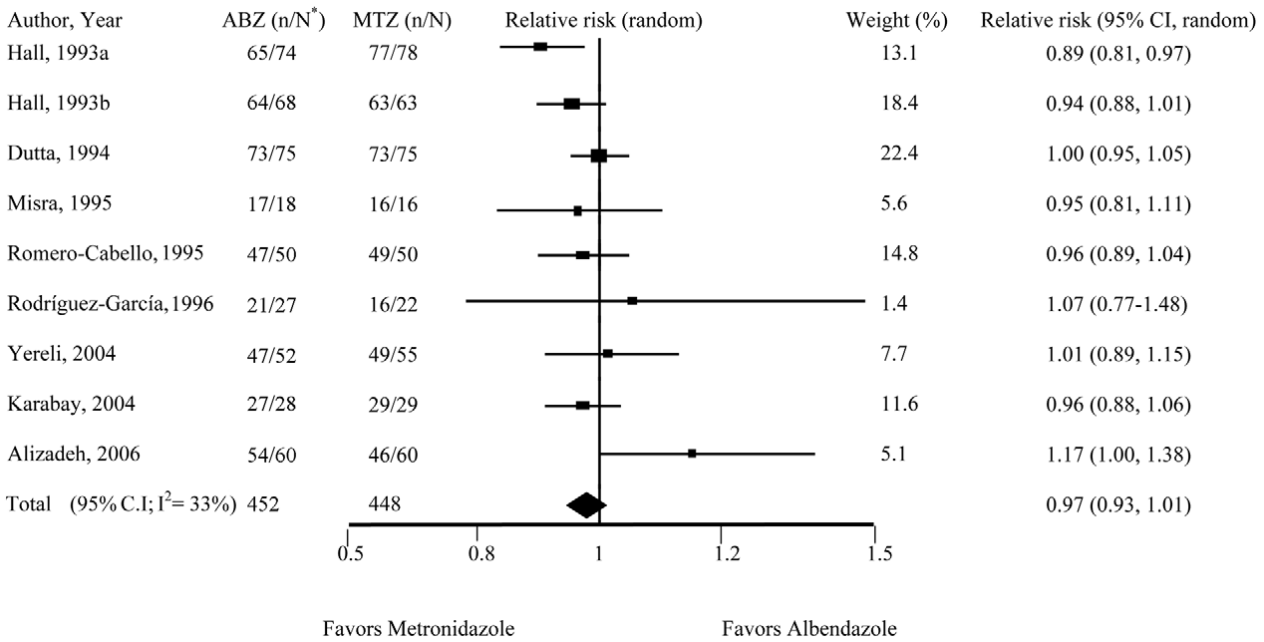
Forest plot showing the effects of albendazole and metronidazole on human giardiasis. Relative risk was calculated for each study separately. n/N = number described as cured over number of participants completing study.

Treatment effects were evaluated as relative risks (RR), estimates that were calculated for each study individually based on the incidence of undetectable infections among those taking metronidazole compared to the incidence of undetectable cases among those taking albendazole. Study-specific RRs were combined using a random-effects model. The study-specific RRs were weighted by the inverse of the sum of their variance and the estimated between-studies variance component [50]. This method calculates the mean difference between the treatment and control groups, with SEM for the difference. There was no statistically significant heterogeneity among these studies using the random effects model (χ^2^ = 11.91, 8 degrees of freedom, *P* = 0.16). An I^2^ of 33% in the current meta-analysis suggests moderate heterogeneity. Results demonstrated no differences between the effectiveness of albendazole compared with metronidazole for treatment of infections with *G. duodenalis* (RR, 0.97; 95% CI, 0.93 to 1.01). When analysis was restricted to trials with a Jadad score of less than 3 (seven trials, 617 patients), inconsistency between the trials was low (I^2^ = 0%), although the overall estimates remained almost constant (RR, 0.99; CI, 0.96 to 1.03). Individual analyses of the eight studies demonstrated that three studies [39], [41], [44], showed a relative risk of greater than 1 for being cured after albendazole therapy (Figure 2). These differences showed that albendazole produced more apparent cures compared with metronidazole. In one study [45], the relative risk was 1 indicating no differences between the effectiveness of albendazole and metronidazole. However, four studies [29], [40], [42], [43], showed a relative risk less than 1 (ranging from 0.89 to 0.96) indicating that metronidazole was more effective (Figure 2). As illustrated in figure 2, the 95% confidence intervals for these studies overlapped to a large degree, suggesting that albendazole and metronidazole are equally effective for treatment of giardiasis.

Further examination of the two-phase study carried out by Hall and Nahar [43] showed that by increasing the duration of therapy with albendazole from 400 mg/d for 3 days (in the first phase) to 400 mg/d for 5 days (in the second phase) the RR increased from 0.89 to 0.94. This suggests that when duration of treatment is similar there is less of a difference between albendazole and metronidazole therapy. Additionally, the efficacy of albendazole in the treatment of giardiasis increased in the second phase of the same study (94.1%) compared with the first phase of the trial (87.8%), implying the need for using albendazole for longer periods of time.

### Publication bias and sensitivity analysis

**Figure 3:**
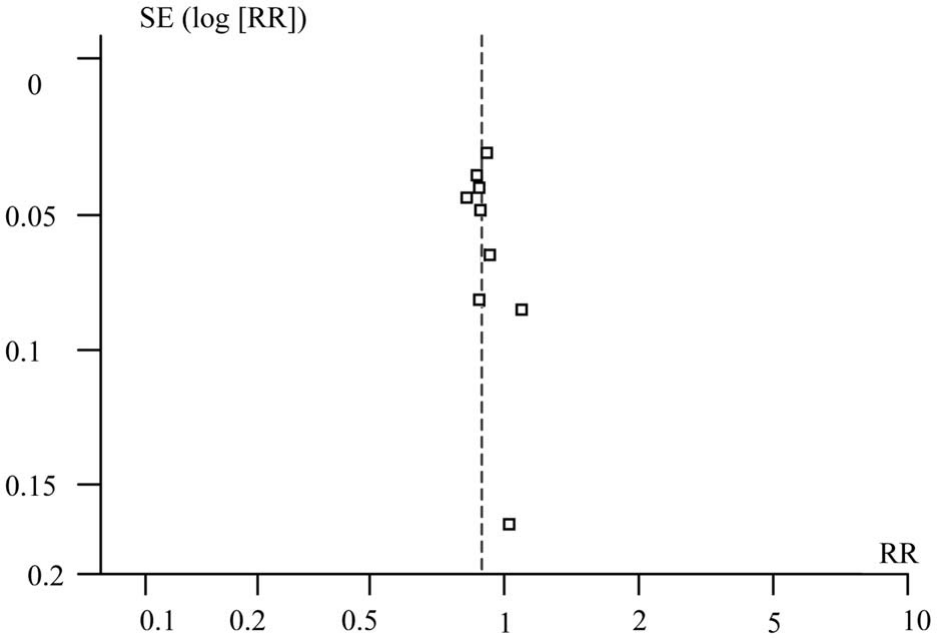
Funnel plots of included studies. The pooled estimate of log-RR for all trials is shown with a dashed vertical line.

Publication bias was examined using a funnel plot. Figure 3 plots the funnel plot of the treatment effects estimated from individual studies on the x-axis (RR) and the standard error of these estimates on y-axis (S.E [log RR]). This analysis shows that the included studies were almost evenly distributed around the vertical axis, providing no evidence of publication bias. To explore further the possibility of heterogeneity due to the use of different outcome measures, we confined our analysis to trials which used the least sensitive methods to detect parasites and then to those that used the most sensitive methods (three methods at the same time). Similarly, we performed an analysis restricted to those studies with clearly defined outcome measures. As seen in Table 4, the overall estimates were equal and the confidence intervals were comparable among these restricted data sets, as well as with the combined meta-analysis values.

**Table 4:** Sensitivity-analysis of the effect of the quality of methods implemented for the measurement of parasitological cure.

Method	No. of Patients	Pooled RR (95% CI)
A	900	0.97 (0.93, 1.01)
B	750	0.96 (0.92, 1.01)
C	524	0.94 (0.91, 0.98)
D	417	0.93 (0.90, 0.98)

A, all eight studies included [29], [39]–[45].

B, one study with unclear outcome measure excluded [45].

C, studies using a single diagnostic method excluded [39], [40].

D, one study which used the most sensitive methods excluded [41].

### Adverse effects

In six studies, side effects related to therapy were absent or were less prominent in the patients receiving albendazole. Only in one study [43] were the reported side effects more evident in patients in the albendazole group compared with those in the metronidazole group (40 cases vs. 7 cases; *P*<0.005). Overall, metallic taste and anorexia were the most commonly observed side effects in patients treated with metronidazole, while loose stools and abdominal pain were more frequent among patients receiving albendazole. Most side effects were transient and no trials were discontinued because of severe adverse effects. In Yereli et al. [41], no side effects were reported in patients treated with either albendazole or metronidazole. The report by Rodríguez-García et al. [44] does not mention if treated children showed any side effects. In order to perform a safety analysis, the two latter studies were excluded from the analysis. Hall and Nahar [43] reported the adverse effects of a two-stage trial, and these were treated as a single trial. Considering all side effects together, 61 of 373 (16.3%) patients treated with albendazole and 82 of 371 (22.1%) of patients treated with metronidazole experienced at least a single side effect. The estimated summary RRs showed that individuals treated with albendazole had a lower risk of adverse effects (RR 0.36) compared with those who took metronidazole, but with a wide confidence interval (95% CI, 0.10, 1.34) that included the null value.

## Discussion

The major finding of this analysis is that when albendazole was given as a single dose of 400 mg/day for 5 days it was as effective as metronidazole in the treatment of giardiasis in humans. Additionally, albendazole had statistically the same safety profile as metronidazole.

Metronidazole has been widely used to treat giardiasis in humans [10], [38], [51], [52], [53], [54], and often causes side effects such as nausea, metallic taste, dizziness and headache [8]. In addition, this drug is a known mutagen in bacteria [55], [56], it is genotoxic to human cells [57], [58] and it has been shown to be carcinogenic in animal models [59], [60]. However, there is no evidence showing metronidazole is also carcinogenic in humans [60]. Typically, metronidazole is administered in doses of 250 mg 3 times a day for 5–7 days for adults and 15 mg/kg 3 times a day for 5–7 days in children. Some clinicians tend to use single-dose regimens, while others like to administer higher dosages for an extended period of time. The latter is problematic in developing countries, as medications are frequently purchased in quantities which represent less than a single day's dose and effective therapies of short duration are preferable [61]. The need for an extended period of time for the treatment of giardiasis again may in part explain the frequent side effects associated with metronidazole therapy. Extended treatment with albendazole also appears to be more effective than shorter duration protocols. However, the once per day regimen would still be preferable to the three times per day required for metronidazole therapy.

A further complication when using metronidazole therapy to treat giardiasis is that the consumption of alcohol should be avoided by patients during systemic metronidazole therapy and for at least 24 hours after completion of treatment [62], [63]. Taking metronidazole and alcohol may result, rarely, in a disulfiram-like reaction (nausea, vomiting, flushing, and tachycardia). It should be noted that the consumption of alcohol by patients was not monitored in any of the studies considered in the current meta-analysis. Alcohol uptake could potentially explain side effects in some patients receiving metronidazole. The lack of placebo-controlled trials makes it difficult to attribute the existence and severity of side effects to either of these two drugs. However, one study that did not meet our inclusion criteria suggested that patients receiving a placebo control presented with minimal side effects [10]. Together, these limitations can potentially restrict the use of metronidazole, in the treatment of giardiasis in humans.

In the trials included in the current meta-analysis, only one study [39] clearly described the inclusion of both adults and children (2–53 yr), while other studies exclusively included either only adults [40] or only children [29], [41], [42], [43], [44], [45]. The inclusion of different age-groups potentially allows us to assess the effectiveness of treatment and to ascertain the extent to which side effects occur in different age-groups. Similarly, including patients with diverse clinical presentations (i.e. asymptomatic, symptomatic; acute, subacute, chronic) in clinical trials could give us the opportunity to evaluate the effect(s) of a given chemotherapy agent/protocol on patients with different clinical manifestations. From the information presented in the articles, it seems that only three articles [41], [42], [44] included both symptomatic and asymptomatic patients, although the clinical status of patients who participated in the study of Hall and Nahar [43] and Dutta et al. [45] was not clear. Including patients from different age-groups and with different clinical presentations in future studies would allow investigators to analyze whether albendazole has a differential effect that correlates to the disease clinical profile and/or age of the patient.

Our analysis suggests that the study designs typically used for evaluating these drugs could be improved. Open-label trials may be suitable for comparing two extremely similar treatments to verify which one is more effective. Only Alizadeh et al. [39] and Hall and Nahar [43] used an adequate protocol for concealing the treatment protocol while determining the parasitological outcome. The six other trials either did not specify or were insufficient in using blinded observers to determine the outcome. Since albendazole and metronidazole may produce certain side effects specific to each drug and since these two drugs may be available in different forms, the use of homogenous therapy regimens and/or using blinded studies may be warranted in future clinical trials.

Several factors may influence the effectiveness of a particular therapy. Nutritional and physiological conditions such as pregnancy and immunodeficiency could potentially alter the effectiveness of a specific drug as shown for other parasitic diseases [64]–[67]. Individuals with “pre-existing” nutritional and physiological (pregnant women) complications were excluded in only two studies among those we have analyzed [40], [45]. Dutta et al. [45] excluded children having grade I and II malnutrition, patients with acute febrile illness and those on long term drug therapy; while Karabay et al. [40] excluded pregnant women and patients with fever from the study. In future studies, it would be desirable to include only patients with no known nutritional, physiological or immunological problems.

Resistance of *G. duodenalis* strains to metronidazole and other drugs has been reported both *in vitro* and *in vivo* [8], [68], [69]. Misra et al. [29] reported a 100% cure rate in groups treated with either metronidazole or albendazole, while the other seven reported an effectiveness of 72.7–100% for metronidazole and 77–97% for albendazole. At least part of the so-called “failure-to-treat” cases might be attributed to the presence of “drug-resistant” strains, a mechanism to which none of the studies referred as a potential reason for treatment failure. The use of different combinations of albendazole with other anti-parasitic agents in future studies may be desirable in order to minimize the risk of the emergence of drug-resistant strains. However, the design of placebo-controlled double blinded clinical trials may help us to better understand the most appropriate regimen(s) and the most suitable chemotherapy protocols.

Some limitations in the current analysis should be considered before making definitive conclusions. First, the small number of trials and patients included in the current analysis (8 studies, 900 patients) led to wide confidence intervals that rendered some of the results inconclusive [70]. Second, publication bias is constantly a potential pitfall in meta-analyses. While we did not try to trace unpublished data for the current meta-analysis, our analysis failed to detect any suggestion of such bias (Figure 3). Third, heterogeneity among studies is another potential limitation to our meta-analysis. It might be argued that differences in the methods used for measuring the outcome of treatment could result in differences in the reported parasitological cure rates, as some combined methods are more sensitive than others. As seen in Table 4, the effect sizes remained fairly constant in these analyses, suggesting that heterogeneity due to diverse outcome measures probably did not adversely affect our analyses. Performing repeated microscopy-based stool examinations on at least two consecutive occasions is sensitive enough to detect up to 95% of infections [71] [72]. This could potentially explain why we did not see any difference among studies employing diverse methods since all the studies required at least two consecutive negative stool examinations before considering the patients cured.

The high rate of side effects from metronidazole therapy for giardiasis, combined with the global emergence of resistant strains, led us to consider the effectiveness of alternative treatments. This meta-analysis revealed that albendazole cures *Giardia* infections with the same effectiveness as metronidazole. However, we were not able to show conclusively, due to limitations of the sample size, that its toxicity profile is more favorable than metronidazole. Therefore, we conclude that larger, double-masked, randomized controlled trials of albendazole and metronidazole with uniform outcome measures are needed to shed light on this important clinical question.
